# (Re)considering Discourses of Risk and Responsibility Through the Lens of Healthism: Interpreting the International Response to a Global Health Strategy for Noncommunicable Diseases

**DOI:** 10.1111/1467-9566.70169

**Published:** 2026-03-12

**Authors:** Tim Brown

**Affiliations:** ^1^ Department of Geography and Environmental Science School of Society and Environment Queen Mary University of London London UK

## Abstract

Public health crises such as the global epidemic of so‐called ‘lifestyle diseases’ are often framed as the failure of individuals to make the right health‐related choices or to take responsibility for managing their bodies in ways that promote the health of present and future selves. Through his early writings on healthism, Robert Crawford was one amongst a number of scholars who documented the emergence of neoliberal logics of self‐care in response to these perceived failings. Although these debates are well covered in the critical literature, less attention has been paid to the ways in which the central tenets of healthism were and are received as ideology travels from place to place. This paper seeks to address this lacuna through a detailed analysis of the discourse surrounding the World Health Organization’s ‘Global Strategy on Diet, Physical Activity and Health’. Chosen for its framing of lifestyle diseases as a global public health problem whose causes are rooted in the spread of the ‘western lifestyle’, the paper argues that a focus on the strategy and the international response to it is revealing for what it tells us about what happens when ideas and theories travel.

## 1 Introduction

As has been widely documented, public health crises, especially but not only those pertaining to ‘lifestyle’ diseases, are oftentimes framed as the failure of individuals to make the right health‐related choices and to take responsibility for managing their bodies in ways that promote the health and wellbeing of present and future selves. To this end, social prescriptions are put in place that may seek to nudge individuals to make what are invariably labelled as ‘healthy choices’ or constrain their capacity to make individually and societally damaging ones through the application of regulatory and other such techno‐social devices. Robert Crawford was one amongst many critical scholars who documented the emergence of this logic of individualised self‐care through his early writings on healthism, medicalisation and risk (see Crawford 1980, 1994). Like others before him, Foucault especially (e.g., Foucault 1976, 1978), emphasis was placed upon the belief that bodies, behaviours, practices and habits were being captured or ensnared in some way or other by western medical science and related cultural, economic and political enterprise. As Crawford (1980, 367) noted, such medicalising processes were associated with an ‘increasing range of social phenomena’ coming into medicine's purview and greater authority was being afforded to its knowledge and practice (see also Conrad and Schneider 1980).

The originality of his early work and arguably the reason for its longevity as a seminal critique of medicalisation lay, at least in part, in its focus on the then nascent holistic health and self‐care movements of 1970s North America. Somewhat counter‐intuitively, Crawford posited that the movement, although emerging as a response to the ‘spectre of a medicalised and medicated society’ (p. 373), was at risk of being equally as medicalising as the western medical authority that it sought to counter. Crawford's argument centred on his belief that these emerging health movements, ‘burdened’ as they were by the ‘ideology of healthism’, continued to situate the problem of ill‐health ‘at the level of the individual’ (p. 374). In making this argument Crawford set out many of the main tropes that would come to be associated with techniques of health‐related governance that are now intimately interwoven into societies reorganised around neoliberal forms of rule. Here, the ‘care’ or ‘cultivation’ of the self, with its emphasis on individual responsibility, is especially significant (see also Cruikshank 1993). Writing in retrospect, he commented *‘[w]hat has become clear in hindsight is that individual responsibility for health*, *although not without challenge*, *proved to be particularly effective in establishing the ‘common sense’ of neoliberalism's essential tenets’* (Crawford 2006, 410. Emphasis in original). Stated more succinctly, ‘[h]ealth talk’ has, in Crawford's reading, become ‘responsibility talk’ (p. 410).

As noted, Crawford was not alone in making these arguments at this time and his focus, like that of his contemporaries (e.g., McKee 1988; Conrad 1992; Lupton 1995; Petersen and Lupton 2000), lay with critically interrogating the individualising tendencies of health and public health movements in the advanced liberal democracies of the West: Crawford's focus was primarily on the USA. In addition to the power exerted by western medicine, the geographical focus of this work was also framed by the then greater prevalence of lifestyle diseases in the aforementioned countries (Brown 2011; Reubi et al. 2016; Manderson and Jewett 2023), the explanatory power afforded to Omran's model of the epidemiological transition (e.g., Reubi et al. 2016; Vaughan 2018), as well as the inclination within western neoliberalism to constitute health as a ‘pan‐value’ (Crawford 1980, 381) or ‘meta‐value’ (Greco 2004, 1) against which people's behaviours and practices were and continue to be judged (see also Conrad 1994). The latter of these was especially important to Crawford's conceptualisation of healthism. On the one hand, he demonstrated that the pursuit of health had come to be ‘emblematic of modern, Western identity’ (Crawford 1994, 1349) and, on the other hand, that a perceived failure to ‘act preventively’ had been reconfigured in moralising terms. As he remarked, the failure to promote one's health was a sign of ‘*social*, not just individual, irresponsibility’ (1980: 380; see also Crawford 1994).

Although Crawford's gaze remained to a great extent spatially fixed on the emergence and development of healthism in the United States, the forms of self‐governance and individual responsibility promoted by healthist ideologies have travelled much further afield. In part, this ‘travelling theory’, to adopt Said’s (1983) terminology, can be attributed to the global mobilities associated with lifestyle diseases and the risk factors for them. Although this mobility is well covered in the critical literature (e.g., Brown 2011; Brown and Bell 2008; Weisz 2014; Weisz and Vignola‐Gagné 2015), less attention has been paid to the ways in which healthist ideologies were received as they travelled from place to place (Vaughan 2018). As Said remarked, ‘what happens to a theory when it moves from one place to another proposes itself as an interesting topic of investigation’ (1983: 230). To this end, the paper seeks to address two important questions. Firstly, it considers how countries outside of the advanced liberal economies of the West responded to their constitution as spaces at heightened risk for lifestyle diseases and, secondly, it explores what reactions there were to the healthist imperatives of an increasingly neoliberal global public health movement. As I argue, although many countries accepted the argument that individuals *should* be made more responsible for their health‐related choices, it was far from clear that they simultaneously accepted that the best solutions to the problem of lifestyle diseases were individualistic ones.

## 2 Tracing Theory: Digital Archival Research

In order to address these questions, this paper focuses on a specific set of policy debates as they played out in the World Health Organization (WHO) in the mid‐to‐late 1990s through to early 2000s. This was a significant moment as it relates to global understanding of lifestyle diseases, which were increasingly being identified as a threat to health and wellbeing globally and not only in wealthy countries (see Aginam 1999; Beaglehole and Yach 2003). As Vaughan (2018) has commented, although international health organisations such as the WHO had been monitoring the growing prevalence of lifestyle diseases or ‘diseases of affluence’ prior to this period, concern for them grew as the evidence of their spread mounted (see also Brown and Bell 2008; Glasgow and Schrecker 2016; Reubi et al. 2016; Herrick 2020, 2022). The anxiety that surrounded the spread of ‘lifestyle diseases’ was amplified further when it was considered more specifically in relation to the nutrition transition, and especially the global obesity epidemic (see Popkin and Doak 1998; Popkin 2002, 2003). Characterised by Elbe as one of several ‘lifestyle timebombs’ (Elbe 2010, 132), the anxiety here was associated with a widely held belief that the globalisation of ‘western lifestyles’ was leading to a double‐, if not triple‐, burden of disease in rapidly urbanising countries, especially those of the Global South (see Brown 2011).

A focus on the debates taking place in the WHO allows for a detailed investigation of the framing of lifestyle diseases and the risk factors for them. It also allows for consideration of the evidence that was being mobilised to promote the need for intervention at a global level and the form that any such intervention should take. Moreover, the organisational structure of the WHO, which retains the regional bodies it inherited from an earlier period of transnational public health (see Birn 2009), allows for an analysis of the differing ways in which countries and regional blocks responded to the development of a global health programme. As a result, it is possible to trace theory, here related to the explanations for and solutions to lifestyle diseases, as it ‘travels’ through an international organisation and to identify what happens when it is grounded in different places. To make this task more achievable, the paper focuses on the debates surrounding a particular event, namely the development of a ‘Global strategy on diet, physical activity and health’ (henceforth ‘Global strategy’) (World Health Organization 2004). As I shall discuss in the following sections, the grounds for such a strategy were established by the WHO in the early 1990s and rapidly developed as the perceived threat from lifestyle diseases became more apparent.

The analysis for this paper is based on digital archival research (see Ventresca and Mohr 2017), which is especially useful for the study of international organisations, such as the WHO, that make documents accessible through online archives. To this end, material relating to the ‘Global strategy’ was identified using the WHO Institutional Repository for Information Sharing (IRIS), which is a searchable database of institutional records. Using a genealogical method, I traced the historical development of the ‘Global strategy’ through associated technical reports, policy documents and the records of the World Health Assembly. Through this approach I was able to identify a significant body of material produced by WHO representatives, as well as by individuals, commercial enterprises, advocacy groups, nongovernmental organisations and national ministries of health all of whom had responded to various consultation exercises. To supplement this research, I traced the response to the ‘Global strategy’ through the Regional Office for Africa (AFRO), which is one of the six regional offices that make up the WHO. This search added significantly to the material collected, particularly in the form of speeches given by the Regional Director for AFRO and transcripts of meetings to discuss the ‘Global strategy’ held at regional events. The paper also draws on the transcripts of speeches given by the Director–General for WHO, Gro Harlem Brundtland, who was responsible for driving the ‘Global strategy’ forward. All documents were downloaded for subsequent analysis.

## 3 Developing a Global Strategy for Health: Building an Evidence Base

In the early years of the 21st century, the World Health Assembly (WHA), the main decision‐making body of the World Health Organization (WHO), endorsed a global strategy to address diet, physical inactivity and health (World Health Organization 2004). Almost inevitably, the declaration which was hailed as a decisive one by the then Director–General of WHO, Dr. Lee Jong‐wook, was reported in the national and international media as a global fight against overweight and obesity. In Britain, for example, Jo Revill and Gaby Hinsliff of *The Observer* newspaper ran the story under the headline, ‘Fit for the future: The world prepares to tackle obesity’ (The Observer, 23 May 2004). Elsewhere, in the Anglophone newspaper media at least, the announcement was reported in a similar fashion, although the language employed did, of course, vary widely: ‘Anti‐obesity plan draughted’ (The New York Times, 22 May 2004), ‘UN's fat attack’ (The Irish Sunday Mirror, 23 May 2004), and ‘UN goes global on battle of the bulge’ (The Australian, 24 May 2004). Although these headlines are revealing of the status of lifestyle diseases in countries long identified as being ‘at risk’ for them, they tell us little about how the proposal for a ‘Global strategy’ was received elsewhere in the world.

To gain understanding of this, I begin by considering the evidence that was mobilised in support of the strategy prior to its announcement in 2004. I do so for several reasons. Firstly, the period surrounding the development of the strategy was one marked by a shift to a metrics‐based approach to global health policymaking (see Adams 2016; Moats 2016; Storeng and Béhague 2017; Tichenor and Sridhar 2020). Secondly, this transition to metrics was, I argue, central to the attempt to persuade countries not typically associated with lifestyle diseases that their populations were at heightened risk for them. Thirdly, as critical scholars have demonstrated over the past decade such numerical representations of reality as the metrics produced in support of the strategy ‘have distinctive effects on knowledge (how things are conceptualised) and on governance (behaviour of actors, policy choices)’ (Fukuda‐Parr and McNeill 2019, 6). To this end, I suggest that an analysis of the evidence is a necessary first step in determining the extent to which some of the central tropes that Crawford associates with the health consciousness of late‐twentieth century North America travelled in parallel with the globalising discourse surrounding lifestyle diseases.

To develop this argument, it is first necessary to unpack the discourse surrounding metric‐based approaches to global health and here I turn to a speech that Brundtland made in her first few months as Director–General. The speech was given to the Regional Committee for Africa, AFRO, which held its 48th session in Harare, Zimbabwe on 31^st^ August 1998. Although a much more wide‐ranging speech than I allow for here, embedded within it were two dimensions that are of particular importance to this paper. Firstly, it was inflected with references to the kind of neoliberal thinking that was reshaping global health at the time, especially the relevance of evidence‐based policymaking for establishing the interconnection between ‘healthy people’ and ‘healthy economies’ (Reubi 2013; Kenny 2015; Adams et al. 2019; Weisz 2022). As Brundtland remarked, ‘[w]e must have the right figures … and the best evidence’ and not only the ‘moral conviction that health is essential’ (Brundtland 1998a). Secondly, Brundtland had little to say on the threat of lifestyle diseases. At this very early stage in her tenure they were conceptualised as a ‘silent epidemic’ and one that would, in time, ‘require a major rethinking of how to succeed in policies of prevention’ (Brundtland 1998b).

The status Brundtland attributed to lifestyle diseases altered, however, by the time that she returned to the African Regional Committee in August 2000. As she stated, ‘the rapid shift of the burden of disease from infectious to noncommunicable diseases will seriously challenge health care systems in the near future and difficult decisions will have to be taken’ (Brundtland 2000a). Brundtland continued, remarking that despite a time lag between ‘exposure to risk and visible outcomes … policy decisions to deal with this shifting burden of disease [are] required *now*’ (Brundtland 2000a. Emphasis added). As this suggests, the apparent rapidity with which lifestyle diseases had emerged as a regional, as well as a global, threat was mobilised by Brundtland to justify to the AFRO delegates the urgency that she associated with developing the ‘Global strategy’. It is not only this message that is important here but also the nature of the evidence that Brundtland was alluding to when making this statement. More specifically, Brundtland's reference to the ‘burden of disease’ signalled the growing influence of the Global Burden of Disease (GBD), as well as metrics‐based approaches to health policy more broadly, on the WHO under her leadership (see Mathers 2020; Weisz 2022). Brundtland had brought Christopher Murray, one of the main architects of the Global Burden of Disease (GBD) project, to the WHO when she arrived in 1998 and he was responsible for embedding this metrics‐based approach within the WHO.^1^

The changing nature of the evidence presented, which was reflected in reports such as the World Health Report 2002 (WHR 2002) (World Health Organization 2002), is important, I argue, because of the impact that it had on the kinds of policy interventions that were legitimated in its name. As stated in the WHR 2002, it had contributed to an understanding of the amount of burden for lifestyle diseases already existing in the world and calculated ‘how much of this present burden could be avoided in the next couple of decades if the same risk factors were reduced from now onwards’ (World Health Organization 2002: xiii). Stated differently, the WHR 2002 quantified the prevalence of risk factors for lifestyle diseases and provided a much more robust and persuasive evidence‐base than had been articulated in previous reports (e.g., World Health Organization 2000). It was largely based upon this evidence that Brundtland, in a subsequent address to the World Health Assembly in May 2002, declared that the world was ‘living dangerously’ not only because people had ‘little choice’ in the decisions that they make but because they were making the ‘wrong choices about consumption and activity’ (Brundtland 2002, 5; see also Brown 2011). Although referring to the global burden here, Brundtland also presented this risk transition in terms that might be viewed as alarmist. As she remarked in the same speech, the report had outlined an ‘intriguing—and alarming—insight’ but ‘[t]he real drama is that they are becoming more prevalent in developing communities’ (p. 4). Therefore, the report, and the discourse that surrounded it, provided a justification for extending already well‐established responses to the management and prevention of lifestyle diseases to the rest of the world (Herrick 2020).

By the time the plans for the global strategy were unveiled by WHO in 2004 an extensive evidence base documenting the main risk factors for lifestyle diseases, including nutrition‐related ones, had been established. The question addressed in the following section is how countries implicated in this ‘risk transition’ viewed the framing of the crisis, here, especially those identified as experiencing a double burden of disease. Here, I turn to the consultation process surrounding the ‘Global strategy’ and address this question to the countries making up the WHO Regional Office for Africa (AFRO). I do so for two reasons. Firstly, because many of the main risk factors identified for ‘high mortality, developing countries’, which includes all the AFRO member states, appear to have more to do with the societal and material constraints people faced than with their making the ‘wrong choices’. As the WHR 2002 report reveals, exposure to ‘unsafe water’ and ‘indoor smoke’, living with nutritional deficiencies (Iron, Zinc, Vitamin A), being ‘underweight’ or having ‘low fruit and veg intake’ were all listed as amongst the top 12 risk factors. Secondly, turning to the consultation process allows me to more carefully trace the passage of the discourse, here through the African regional office, and to explore the ease with which the central tenets of the ‘Global strategy’ travelled.

## 4 Responding to a ‘World at Risk’: A Story From the Global South

By way of providing some background, it is worth noting that the consultation surrounding the ‘Global strategy’ was not the starting point for discussion on the topic in the African region. As the Director of the African Regional Committee remarked at the Ouagadougou meeting attended by Brundtland in August 2000, the need for action on lifestyle diseases had been raised by their members at the WHA for well over 3 decades at this point and the WHO had frequently been requested to ‘intensify’ its prevention measures in the region (WHO Regional Office for Africa 2000, 3). Perhaps because of this longstanding regional awareness of the problem, the Director adopted a similar perspective to Brundtland when it came to describing the contemporary situation. As he declared, ‘there is currently a rapid epidemiological transition’ which would ‘become significantly apparent in the coming decades if nothing is done about the situation’ (WHO Regional Office for Africa 2000, 1). However, the Director struck a subtly different tone when it came to providing a prescription to the problem, one that explicitly highlighted the precarious state of the healthcare system across the region. As he remarked, existing health systems were ‘inadequate to deal with NCDs’, they relied on services that were too ‘expensive’ had limited ‘coverage and impact’ and could not ‘guarantee accessibility and equity when costs are virtually unbearable for both health systems and households’ (p. 1, 3). The health system, he argued, was simply ‘not adequately prepared’ and the solution lay in developing a ‘comprehensive and coherent community‐based approach’ (p. 3).

As this suggests, there is a tension at play here. On the one hand, there was qualified agreement across the region that a nutritional and epidemiological transition was underway and that there was a need for a collective response to it. This point was articulated not only in this meeting of the African Regional Committee but throughout the regional response to the development of the ‘Global strategy’. For example, at the 55th meeting of the World Health Assembly in May 2002, where Brundtland had suggested to delegates that the world was living dangerously, representatives from AFRO member states broadly agreed with her sentiment. The Namibian representative, speaking on behalf of countries from the Southern African Development Community (SADC), declared, ‘[w]e are faced with common challenges … [including] the rapidly increasing prevalence of noncommunicable diseases’ (World Health Organization 2003a, 71). Similarly, the Kenyan representative reported, ‘[a]ll of us gathered here will acknowledge that in most parts of the world, noncommunicable diseases, which are primarily preventable, have become a major epidemic’ (World Health Organization 2003a, 140). Echoing the others, the Ghanaian representative called for a ‘crusade of the same magnitude as has been waged against communicable diseases’ (World Health Organization 2003a, 88). Declarations to this effect were also made following the announcement of the ‘Global strategy’ in May 2004 (see World Health Organization 2004).

Viewed from this perspective, Brundtland's approach to evidence‐based policymaking could be regarded as an extremely effective and persuasive one. However, despite this apparent agreement with the idea that a risk transition was underway, there was also significant divergence in the response to the framing of the problem of lifestyle diseases among the countries represented by AFRO. I focus on the nature of this counter‐discourse because I contend that by considering it we are able to witness how ideas, here relating to healthism, are challenged, amended and recast as they travelled from the spaces of WHO's central offices in Geneva to those of its regional centres. To achieve this, I delve deeper into the consultation which began in earnest in March 2003 and lasted for a further 18 months. Framed by a technical report jointly produced with the Food and Agriculture Organization of the UN (FAO) (World Health Organization/Food and Agriculture Organization of the UN 2003), as well as by a consultation document produced by the WHO Noncommunicable Diseases and Mental Health Cluster (NMH), the process was devised to alert as broad a sector of society as possible to the ‘health problems caused by unhealthy diets and physical inactivity’, of their ‘devastating social and economic outcomes’ and about ‘proven [evidence‐based] prevention interventions’ (World Health Organization 2003a, 2). As the consultation document declared, the aim was to ‘inform, convince and mobilise’ stakeholders as well as to establish any ‘regional differences, common concerns, or global consensus’ (World Health Organization 2003b, 3). It was these documents that framed the consultation that took place with members of the Regional Office for Africa in Harare, Zimbabwe over 3 days in March 2003.

Following the same format as other regional consultations,^2^ the meeting was attended by representatives of 14 out of a possible 47 African member states, a range of technical experts from within and without the continent, including one from NMH, as well as members of the WHO secretariate and other UN agencies with offices in Harare (FAO and UNICEF). Opened by Dr Mohammed Belhocine, the Director of the Division of Noncommunicable Diseases for AFRO, emphasis for the meeting was placed on the need to develop a ‘clear strategic vision’ and ‘concrete and concerted action’ as well as upon the desire for members to share their experiences and to ‘advocate for the adoption and successful implementation of the global strategy’ (World Health Organization 2003, 6). Beyond this, the consultation process involved WHO representatives at the meeting seeking to reinforce key messages that underpinned the need for such a strategy. For example, the suggestion that the ‘incidence of NCDs is rising rapidly’ or that ‘80% of the NCD burden is now found in the developing world’ (World Health Organization 2003, 7, 8) reinforced the belief that lifestyle diseases are a global threat to health rather than only a national or regional one. This repetition of core statements about the present and future health consequences of lifestyle‐related disease burden was itself supported by statements about the strength of the evidence base, the significance afforded to risk factor analysis and the interconnected belief that NCDs are ‘preventable diseases’ that require only ‘relatively modest behavioural changes’ to promote ‘swift and dramatic changes in population health’ (WHO 2003c, 8).

As this suggests, the WHO consultation was a part of a wider strategy to diffuse and normalise ideas relating to the mobility of the risk factors for what were previously understood as western lifestyle diseases. Read in this way, the discourse presented by the WHO secretariate is suggestive of the ways in which Crawford argues a new health consciousness has taken shape in the 21st century. As he commented, the ‘diffusion of health knowledge, with its ever‐expanding list of dangers, has many sources’ (2006: 415). Amongst these, he lists the ‘acceleration’ of epidemiological research, the ‘identification and politicisation’ of hazards, and the expansion of technologies for detecting and monitoring risk (2006: 415). All of these elements apply here. However, despite the deployment of this global health assemblage surrounding lifestyle disease there was resistance to this framing amongst the health officials present at the AFRO consultation. For example, in their individual country submissions the delegates offered up alternatives to lifestyle diseases as the primary public health issues in their countries. To this end, the Cameroonian delegate reported that ‘HIV/AIDS and other sexually transmitted diseases are the major public health concerns’ for their country, the Algerian, like the Tanzanian, that their country's health problems were ‘largely related to communicable diseases, aggravated by nutritional deficiencies’, and the Kenyan delegate focused on the causes of undernutrition such as ‘poverty, climate‐related issues such as drought’ as well as on ‘communicable diseases’ (WHO 2003C, 11–12).

These counterarguments to the WHO framing, although subtle, were also evident at other stages of the consultation process and in other spaces associated with it. As an illustration of this, the G77 group of countries submitted a strong rebuttal of the technical documents underpinning the consultation at a meeting with the FAO Committee on Agriculture held in February 2004. As they stated, ‘it is the view of G77 that the WHO Technical Report Series 916 fails the test of scientific rigour, objectivity and fairness’ (G77 2004). Going further, they accused the report of being ‘biased’, ‘arbitrary’, suffering from ‘methodological flaws’, ‘tinted towards selectivity’ and having a ‘strong impressionistic tone’. Perhaps more damningly, they stated that the WHO ‘obsession’ with obesity undermined the more serious ‘problem facing humanity, namely the presence of 2 billion people all over the world suffering from under‐nutrition and micro‐nutrient deficiency’ (G77 2004, 2). Similar, arguments were made in the individual submissions of countries from across the region. The Mauritian government, for example, drew upon the G77 submission but utilised it to declare the basis upon which they would support the ‘Global strategy’. As the text from that submission reveals, this extended well beyond the kind of risk‐factor oriented and targeted approach that WHO was advocating for:

‘G77 are open to proposals for changes in the prevailing diets, *provided that such proposals are science based, supportive of a better balance in total energy intake, undamaging to national food security, economically affordable, sensitive to cultural heritage, causing no harm to the prevailing food production system, food processing and food trading practices, environmentally friendly and which protect the rights of farmers, herdsmen, fishermen and foresters. In addition, such proposals must have the support of the wider scientific community*’

(Mauritius Mission to the United Nations Geneva 2004. Emphasis in original)

## 5 Promoting Healthy Choices: Beyond Individual Responsibility?

In the previous two sections, I focused upon the evidence constructed around the ‘Global strategy’ and explored the response to it through an analysis of the discourse surrounding the consultation process relating to one of the WHO's regional offices. As this analysis suggests, there was a general acceptance that lifestyle diseases were an emergent threat to health and recognition that the risk factors for them had travelled. However, there was resistance too. In part, this resistance stemmed from concerns with the evidence presented in the consultation documents which drew on a range of sources and not only the metrics produced by the World Health Report 2002. It is also possible that the issues raised related to a perceived over‐interpretation of the evidence presented in the report, as much as the evidence itself. To this end, although country representatives appeared willing to accept that lifestyle diseases and the risk factors for them were on the rise, they seemed less willing to believe that the resulting epidemiological and risk transition shared the same degree of importance as the many other issues faced by countries in the region (see Vaughan 2018). For most, it was not the ‘drama’ that Brundtland suggested it was and the emphasis placed on obesity was seen by some as representing more of an ‘obsession’.

In this section, I move on to consider the mobilisation of ‘healthy choice’ within this discourse and do so for several reasons. The first relates to promoting an understanding of the ways in which ‘healthy choice’ and responsibility were discussed in the ‘Global strategy’ and the differing ways in which responsibility was distributed. Turning here to the strategy document itself, there was an acknowledgement that responsibility for the globalisation of risk factors for lifestyle diseases was shared among multiple stakeholders, ranging from governments, international organisations, the food and agricultural industries to communities and individuals. Although responsibility was shared, the strategy distributed the power to affect change in very different ways. For example, those identified as having control over the potentially toxic environments people inhabit were tasked with the responsibility to enact change that ‘*empowers and encourages* behaviour changes’ and enables people to ‘make *positive*, *life‐enhancing* decisions on healthy diets’ (2004: 7. Emphasis added). To this end, governments were advised of their responsibility to communicate, inform, regulate, surveil and evaluate all aspects of public policy that enable ‘healthy choices’ (World Health Organization 2004, 15). Similarly, civil society and NGOs were advised of their role in ‘influencing individual behaviour’ (p. 20) and the private sector was encouraged to ‘promote’, ‘limit’, ‘consider’, ‘provide’, ‘practise’ and ‘issue’ a plethora of actions in their capacity as ‘responsible employers and as advocates for healthy lifestyles’ (p. 21).

That responsibility was distributed in this way should come as little surprise. As mentioned previously, Crawford was among many scholars who interrogated the individualising of responsibility for health and its alignment with the neoliberal logics that informed the emerging health consciousness of the mid‐to‐late‐twentieth century. As he remarked, ‘health consciousness and individual action to protect and improve health have become integral to the quest for security’ (Crawford 2004, 505). Although Crawford was primarily concerned with tracing this discourse as it related to people's everyday health beliefs and practices, he also paid close attention to the role played by differing forms of governmental knowledge in promoting this understanding. Here, especially, he highlighted the importance of health promotion which cultivated the message that ‘contemporary life is lived in the danger zone’ and that ‘lifetime prevention’ should form an ‘essential part of everyday practice’ (Crawford 2004, 509). Although not stated, this reading chimes with Foucault‐inspired critiques of health promotion which regarded it as a form of neoliberal governmentality (LeBesco 2011; Glasgow 2012). Therefore, it is not only the responsibility of experts to communicate knowledge associated with health‐related risks but also to help establish the health‐promoting environments within which individuals and communities can act. As Brundtland stated at the Fifth Global Conference on Health Promotion, held in Mexico City in June 2000, ‘for people to have the power to be healthy, they first need knowledge’ and they must be ‘empowered to make the healthy choices for themselves’ (Brundtland 2000b).

As this suggests, despite a stated ambition to shift responsibility and blame away from individuals and communities, the imperative remains with them to make healthy choices once the environments they inhabit are ‘fixed’ (Mayes 2015; Herrick 2020; Herrick and Bell 2022). This perspective was widely reflected in the submissions to the consultation for the ‘Global strategy’. For example, in their submission, the United States declared that individuals were responsible for being ‘more active’ and for making ‘better and healthier choices for their family’, industries for providing and promoting ‘healthier choices for customers’ and governments for ensuring ‘the public has accurate, science‐based information needed’ (US Department of Human and Health Services 2004). Where this delineation of responsibility more or less matched the one set out in the WHO strategy, in other responses significantly greater weight was placed upon the role of individuals, with the Australian response declaring that governments cannot ‘regulate to overcome poor dietary choices’ (Australian Department of and Health and Ageing 2003). A similar view was held by the Indian response which asserted that ‘[f]ood cannot be treated on same footing as tobacco. The decision to eat or not to eat a food rests with an individual’ (Indian Department of Health and Family Welfare 2004).

Returning to an earlier cited quote from Crawford (2006), these responses, which were broadly mirrored across other member states, suggest that the ‘common sense’ of neoliberalism appears to have travelled without much hindrance when ideas of the individual responsibility for health and healthy choice were evoked in the ‘Global strategy’. Although this may appear so, it is also important to acknowledge, following Said (1983), that theory meets resistance as it travels and is adapted and changed as it does so. On the specific question of applying neoliberal logics to public health discourse, Bell and Green (2016) are especially instructive. As they argue, there are parallels and divergences in the framing of neoliberalism's central tenets, and we need more ‘nuance and specificity’ in our accounts of it (2016: 241–2). Bearing this in mind, there was also significant nuance in how countries framed responsibility and choice. In many instances, governments or their representatives sought to qualify or place limits on the boundaries of individual responsibility. Thus, individuals were identified as being responsible but only within environments that make healthy choices the ‘easier’ ones, the more ‘accessible’ ones, or, in the words of the Danish Ministry of the Interior and Health, the ‘natural’ ones (2004). Other country representatives were even more forthright in attributing blame to the societal contexts that shape an individual's capacity to make healthy choices: as the Romanians commented, ‘the poorest member of societies are actually encouraged to make unhealthy choices because the least healthy choices are the only ones they can afford (Romanian Ministry of Health, n.d.). The implication in these responses was that the social and policy environments within which healthy choices are made must be reconfigured if individuals are to be ‘empowered’.

This questioning of the evidence base and positioning of lifestyle diseases relative to a much broader range of concerns than those presented in the WHO consultation document was also reflected in the priorities for action set out by delegates taking part in the consultation with the Regional Committee for Africa in Harare. Responding to the suggestion that governments may need to ‘simultaneously address *unbalanced nutrition*, *over‐nutrition* as well as *under‐nutrition*’ (World Health Organization 2003b, 5. Emphasis in original), the priorities that emerge and the framing of them spoke more directly to the social and material constraints that shaped the latter. Although it was noted that there was a need to ‘draw attention to the true complexity of the [nutrition] situation’ and to ‘highlight the need to develop comprehensive nutrition policies that encompass all forms of malnutrition’ (World Health Organization 2003b, 14), the priorities pointed to the broader range of issues outlined in the previously discussed G77 response—that is, those relating to food and nutrition security, economic development and the regulatory and trade environment. Where the former was concerned with issues of food availability and access, especially for ‘those who are poor [and] cannot afford sufficient food or enough food of the right quality to provide a balanced diet’, questions of development related to the whole gamut of issues that most countries in the region faced. These ranged from poverty, unemployment and underdevelopment to the absence of the necessary infrastructure to supply clean and safe water; as the consultation document noted, ‘all affect the ability of people to make healthy dietary choices’ (World Health Organization 2003b, 15). Further, it is important to acknowledge that the concern for lifestyle diseases, and here especially the issue of overnutrition as a risk factor for them, was consistently situated in a subordinate position to undernutrition. As the delegates set out, ‘[u]ndernutrition remains the *dominant* feature in Africa, and nutrition policies and planning in Africa overwhelmingly focus on this aspect of malnutrition’ (World Health Organization 2003b, 14. Emphasis added).

## 6 Conclusion

In this paper, I have turned to a significant moment in the development of an international response to an emerging global crisis and one that has helped to shape the past two‐decades or more of public health intervention. Specifically, I focused on the discourse surrounding the ‘Global strategy’ developed by the WHO in the early 2000s because it provided an opportunity to engage critically with the individualising tendencies that are often associated with public health responses to lifestyle diseases and their causes. In particular, I was interested to understand how countries responded to the evidence presented to them about their risk status as well as to the ways in which responsibility for the emergence of the crisis was distributed. In asking such questions, this paper seeks to contribute to critical reflection on Crawford's (1980) seminal paper on healthism, as well as to subsequent analyses of the individualism that remains inherent within public health interventions that target lifestyle diseases, in several important ways. As I argue, the language of the strategy is such that it appears to confirm the belief that responsibility for health has been, and continues to be, individualised in ways that correspond with Crawford's rendering of healthism. However, as the strategy came into being and consultation surrounding it took place, the evidence presented by the WHO appears to have failed to persuade many of its members that lifestyle diseases were, in fact, their greatest health‐related challenge (see Vaughan 2018). It is on the implication of this point that I conclude.

With regard to the key findings, the paper draws on the discourse surrounding the ‘Global strategy’ to make two key arguments. The first of these relates to the nature of the evidence provided to justify the strategy and the response to it from across a range of countries, including those from one of the regions identified as being at heightened risk, namely continental Africa. As the paper illustrates, the evidence for the strategy evolved during this period as the recently arrived Director–General of the WHO, Gro‐Harlem Brundtland, turned towards the metric‐driven approach of the GBD (Weisz and Vignola‐Gagné 2015; Adams 2016). Generally regarded as signalling a paradigmatic shift in global health, the metrics outlined in the World Health Report 2002 established a framework for ‘objectively identifying’ global epidemiological priorities for lifestyle diseases and cost‐effectively responding to them (Weisz and Vignola‐Gagné 2015, 513). However, the evidence‐base that was mobilised in the consultation surrounding the development of the strategy was met with resistance. Focussing primarily, although not exclusively, on the response of countries from the WHO Regional Office for Africa, I illustrate how the evidence underpinning the strategy was critiqued for its lack of scientific rigour, bias, selectivity and subjectiveness. The significance of this here is that the perceived limitations of the evidence base appeared to temper the response of countries to the ‘Global strategy’ and perhaps resulted in a rather tepid response to it. Although there was acknowledgement that lifestyle diseases were a global concern, this did not easily translate into their being prioritised in contexts where other health‐issues remain(ed) more important.

A second, and closely related, dimension of the paper was to explore how ideas of responsibility, and especially individual responsibility, were engaged with by the stakeholders responding to the consultation. This is, of course, significant in the context of a paper that seeks to engage with Crawford's (1980) work on healthism and especially his argument surrounding the various ways in which responsibility for health has been individualised. This paper's contribution to understanding here is to suggest that a degree of caution is required when seeking to understand and interpret the extent to which a paradigm that appears hegemonic in one place travels to and is adopted in another. To an extent the authors of the global health strategy were conscious of this, stating that national strategies needed to be ‘culturally appropriate’ and responsive to ‘changes over time’ (World Health Organization 2004, 10). However, this acknowledgement of cultural differences and the dynamism it suggests does not equate to a withdrawal from a perspective which arguably reanimates Crawford's contention that healthism ‘adopts an independent strategy for personal enhancement against the external force and internal weaknesses which assault well‐being’ (1980: 381). How else are we to interpret the confirmation of a strategy that emphasises the belief that the pathway to health lies in promoting enabling environments that empower individuals to make healthy choices? Afterall, this emphasis on the individual responsibility for making healthy choices was as central to the inauguration of the WHO global strategy for NCDs in 2004 as it remained in the most recent confirmation of it at the UN High Level Meeting in 2018 (UN General Assembly 2018). However, the analysis presented in this paper highlights that although ‘ideas and theories travel’, as Said (1983) argued, their ‘transplantation’ and ‘transference’ is complicated by the conditions into which they arrive. Although individual responsibility for making healthy choices was acknowledged as important, for many of the countries responding to the WHO consultation neither this issue nor the problem of lifestyle diseases was their main concern. In their place, they encouraged the WHO to look further upstream and to the conditions of existence that Crawford argued must be addressed if a ‘viable health strategy’ is to be genuinely achieved (Crawford 1980, 385).

## Author Contributions

**Tim Brown:** conceptualization, data curation, formal analysis, writing – original draft, writing – review and editing.

## Conflicts of Interest

The author declares no conflicts of interest.

## Data Availability

Data sharing not applicable to this article as no datasets were generated or analysed during the current study.

## Endnotes

^1^
Murray was appointed as Executive Director of the Evidence and Information for Policy Cluster at the WHO between 1998–2003.

^2^
Comparison of the six regional consultations reveals that the same opening text was reproduced in all of the reports, with only some minor amendment to account for the differing regional contexts.
